# Response to “Assessment of Renal Function in Transgender Patients With Kidney Disease”

**DOI:** 10.1177/20543581211020178

**Published:** 2021-06-04

**Authors:** David Collister, Nathalie Saad, Emily Christie, Sofia Ahmed

**Affiliations:** 1 Department of Medicine, Division of Nephrology, University of Manitoba, Winnipeg, Canada; 2 Chronic Disease Innovation Center, Seven Oaks General Hospital, Winnipeg, MB, Canada; 3 Department of Medicine, Division of Endocrinology, University of Calgary, AB, Canada; 4 Department of Medicine, Division of Nephrology, University of Alberta, Edmonton, Canada; 5 Department of Medicine, Division of Nephrology, University of Calgary, AB, Canada; 6 Libin Cardiovascular Institute, University of Calgary, AB, Canada

We thank Jue et al for their comments regarding the assessment of renal function in transgender patients with kidney disease. We agree that any evaluation should include consideration of a patient’s anatomy, medical history, medications, and laboratory values. We also agree that the measured glomerular filtration rate (mGFR) by either 24-hour urine for creatinine clearance or an alternative method is necessary if an accurate and precise measure of GFR is needed for clinical decision-making. However, mGFR has its own limitations and may not be feasible to be assessed or repeated at all clinical encounters. Similarly, 24-hour urine collections are often inaccurate even in tertiary care settings.^1^ Thus, we recommended using the range of estimated GFR (eGFR) using both sex at birth and current gender identity for all transgender patients, including those treated with long-term gender affirmation hormone therapy (GAHT). Ideally, this value could be confirmed by mGFR with eGFR used longitudinally. These ranges must be taken with consideration of the patient’s estimated muscle mass, which may be evolving over time with the use of GAHT and other interventions.

We understand their concerns about extrapolating data from small case series and cohort studies with heterogeneous populations and interventions. Our review only included 9 studies, whereas that of Webb et al^2^ included only 4 studies, 2 of which are cited in our review. However, their review also highlights other pharmacokinetic differences between sexes and genders including absorption, volume of distribution, protein binding, and hepatic metabolism which are relevant to transgender persons and their clinicians. There is clearly a need for large population-based cohort studies and a systematic review addressing the assessment of kidney function and the impact of GAHT on kidney function in transgender persons.^3^ Finally, the development and validation of eGFR equations,^4,5^ specific to transgender persons, should be a research priority, but this will require collaboration across institutions given at present, the relative rarity of the intersection of transgender persons, and chronic kidney disease.
